# A Descriptive Study of Nosocomial Infections in an Adult Intensive Care Unit in Fiji: 2011-12

**DOI:** 10.1155/2014/545160

**Published:** 2014-09-17

**Authors:** Keshni Naidu, Ilisapeci Nabose, Sharan Ram, Kerri Viney, Stephen M. Graham, Karen Bissell

**Affiliations:** ^1^College of Medicine, Nursing & Health Sciences, Fiji National University, Suva, Fiji; ^2^Colonial War Memorial Hospital, Ministry of Health, Suva, Fiji; ^3^Public Health Division, Secretariat of the Pacific Community, Noumea, New Caledonia; ^4^National Centre for Epidemiology and Population Health, Research School of Population Health, Australian National University, Canberra, Australia; ^5^International Union Against Tuberculosis and Lung Disease, Paris, France; ^6^Centre for International Child Health, Department of Paediatrics, University of Melbourne and Murdoch Children Research Institute, Royal Children's Hospital, Melbourne, VIC, Australia; ^7^The University of Auckland, Auckland, New Zealand

## Abstract

Nosocomial infections in an intensive care unit (ICU) are common and associated with a high mortality but there are no published data from the Oceania region. A retrospective study in Fiji's largest ICU (2011-12) reported that 114 of a total 663 adult ICU admissions had bacteriological culture-confirmed nosocomial infection. The commonest sites of infection were respiratory and bloodstream. Gram negative bacteria were the commonest pathogens isolated, especially *Klebsiella pneumoniae* (extended-spectrum *β*-Lactamase-producing), *Acinetobacter,* and *Pseudomonas* species. Mortality for those with a known outcome was 33%. Improved surveillance and implementation of effective preventive interventions are needed.

## 1. Introduction

Health-care-associated (or nosocomial) infection is a major problem in hospitals worldwide and the prevalence is two- to threefold higher in developing countries compared to Europe or USA [1, 2]. The incidence is particularly high in intensive care units (ICUs) compared to non-ICU wards in the hospital as ICU patients have a range of severe comorbidities and the use of invasive devices during their management is very common [3]. Reports from a range of ICU settings including those in developing countries consistently show a high burden of device-associated nosocomial infections [4–6]. Nosocomial infections are caused by a wide range of pathogens, and ventilator-associated pneumonia and central line infections are common sites of infections and are associated with high mortality—as high as 50%. Nosocomial infections are associated with an increase in crude mortality, length of stay in ICU, and hospital costs [7–10].

There are no published data reporting the prevalence of nosocomial infections in Fiji. Such information is required to describe the current epidemiology and to improve infection control practices in the adult ICU. This retrospective study aimed to describe bacteriological culture-confirmed nosocomial infections in Fiji's largest adult ICU.

## 2. Materials and Methods

### 2.1. Study Design and Setting

This was a retrospective, descriptive study of bacteriological culture-confirmed nosocomial infections in an adult ICU in 2011 and 2012. The study was conducted at the Colonial War Memorial Hospital (CWMH) in Suva, Fiji. The adult ICU ward at the CWMH has 6 beds and is the largest ICU ward in Fiji. There is a separate ICU ward for children and neonates (0–14 years). The adult ICU provides care for both ventilated and nonventilated patients and a mix of medical and general surgical patients. Patients may be admitted directly from the community or from other hospital wards.

### 2.2. Study Population

The study only included patients with clinical features of invasive sepsis who had a bacterial pathogen isolated on culture from at least one specimen taken from the patient more than 48 hours after admission to the ICU, that is, those with clinically suspected nosocomial sepsis that was microbiologically confirmed. When the onset of invasive sepsis is clinically suspected on the basis of the patient developing evidence of a systemic inflammatory response syndrome (SIRS, see Box 1), samples from venous blood, body sites, and devices are sent for bacterial culture. The attending clinician selects the samples to be sent as clinically indicated on the basis of the likely site of infection. Patients admitted to the ICU with sepsis were not included unless they developed new signs of SIRS (Box 1) more than 48 hours after admission. Patients with suspected sepsis without microbiological confirmation were also not included.

Blood cultures were processed using the BACT/ALERT 3D system (BioMerieux, Marcy L'Etoile, France) and incubated at 36°C for 7 days. If a blood culture bottle was flagged positive, a Gram stain was performed on one drop of the culture fluid. Subculture was performed using selective and nonselective agar depending on the Gram stain result. Gram negative organisms were identified using the Microbact Identification Kit (Oxoid, Basingstoke, UK).

Data were sourced from the infection control surveillance records and the ICU patient records with cross-checking between these records and the microbiology laboratory records. Data collected for all ICU admissions for 2011-2012 included numbers of admissions, devices used, and number of days the devices were used. The patient name was used for cross-checking between registers but not included in data entry or data storage.

Data were collected in a structured proforma using a unique identification number and the following variables were entered: age, date of admission, date of specimen collection, length of stay in ICU, and bacterial pathogens isolated and from which specimen the pathogen was isolated. Respiratory specimens include those that were taken from the respiratory tract—usually aspiration of endotracheal or tracheostomy tubes from ventilated patients—or pleural aspirates while blood stream infections include those taken from peripheral blood cultures, those samples taken through a venous cannula device such as a central venous line and catheter tips.

Data were double entered into EpiData version 3.1 (EpiData Association, Odense, Denmark). The rate of nosocomial infections was calculated by dividing the number of patients with nosocomial infections by the total number of patients in the ICU during the same study period. Numbers of isolates per site of specimen are presented.

## 3. Results

There were 663 patients admitted to the ICU during the two-year study period. This represented 2891 total patient days of admission during which patients were ventilated for 2175 days. Of the 663 admissions, 114 (17%) developed culture-confirmed nosocomial sepsis.  Table 1 lists the characteristics of the study patients. The majority of the patients had prior admission to other wards before admission to ICU. Males were more commonly represented than females and almost all patients had been mechanically ventilated with a median period of 8 days of ventilation.

Table 2 lists the bacterial pathogens isolated and the sites from which they were isolated. The largest proportion was from respiratory tract specimens. There were 437 isolates from 114 patients and so most patients had isolates cultured from multiple sites. Of patients, 66% had isolates from a respiratory specimen (endotracheal tube or pleural fluid), 49% from a urinary specimen (indwelling catheter or clean catch), 67% from a blood specimen (peripheral or central line), and 41% from a surgical site (wound swab or surgical drain). Gram negative bacteria such as* Klebsiella pneumoniae*,* Acinetobacter* species, and* Pseudomonas aeruginosa* were the commonest isolates.* K. pneumoniae* (extended-spectrum *β*-Lactamase (ESBL) producing) was isolated from blood and urine in 21% of patients simultaneously. Coagulase-negative staphylococci were most commonly isolate from blood.* Pseudomonas aeruginosa* was the commonest isolate from surgical site specimens.

Outcome was known in 84% (96/114) patients and 40% (38/96) with known outcome died in ICU (Table 1). Of 63 patients with a bacterial isolate from the blood and a known outcome, 21 (33%) died, compared to 50% (16/32) among those with a known outcome but without a bacterial isolate from the blood. Of those that died, 55% (21/38) had a blood stream infection.

## 4. Discussion

This study provides original descriptive data from Fiji of common nosocomial bacterial infections in ICU. A recent global systematic review of the published literature on the burden of nosocomial infections in developing countries did not identify previously published data from the Pacific Island region [1]. The commonest sites of infection were respiratory tract and bloodstream infections, and Gram negative bacteria were the main pathogens isolated from ICU patients. These findings are consistent with other studies from developing countries [1, 4–11].

Mortality was very high among these ICU patients with nosocomial infections. High mortality has been previously reported from developing countries ICUs [6, 10]. It is difficult to ascertain the additional attributable risk of nosocomial infection to mortality in these patients and settings as overall mortality is high in ICU patients and length of stay of ICU patients is linked with the comorbidities that the patients have prior to admission to ICU. The average length of stay for our patients was 4 days but with a wide range of up to over 30 days—such as for patients with trauma or Guillain-Barre syndrome. Patients in the ICU were ventilated for the majority of the time spent in ICU, on average for 75% of their stay. However, we were unable to determine relationships between nosocomial infection and outcomes such as mortality and length of stay in our retrospective study. A previous study estimated that ventilator-associated pneumonia, which is one of the commonest ICU-related nosocomial infections, was associated with a 14% increase in risk of mortality and a prolonging of the ICU stay by two days [10].

In this study, Gram negative bacteria were the predominant pathogens isolated with* Klebsiella, Acinetobacter,* and* Pseudomonas *the commonest isolate. These pathogens are commonly isolated from patients in ICUs and the prevalence of pathogens can change over time [3–6, 12–14]. There was a recent outbreak of* Acinetobacter *in the ICU of CWMH during the study period (August–December 2012). Gram positives were less prevalent with the commonest being coagulase-negative staphylococci and it is difficult to know whether this is a pathogen or skin contaminant [13]. It was most commonly isolated from blood specimens that are often taken through intravenous lines. Notably, methicillin-resistant* Staphylococcus aureus *was not commonly isolated. Nosocomial pathogens are frequently resistant to multiple antibiotics. We do not present antibiotic susceptibility data except to note that ESBL-producing* Klebsiella *was very common.* K. pneumoniae* is well recognized to have emerged as important ESBL-producing bacteria, and the other common bacteria found in this study such as* Acinetobacter *and* Pseudomonas *can also be ESBL-producing [13].

This study has a number of important limitations. It is a retrospective study that relies on previous data records for which accuracy and completeness cannot be validated. However, protocols and recording are structured and have been used for infection control purposes in the CWMH for many years. Data on the use of all devices over the study period were not available and so rates of nosocomial infection associated with specific device utilization over time could not be calculated. The data on outcome were also incomplete.

## 5. Conclusion

Despite these limitations, this study provides important baseline data that strongly suggest that infection control practices could be improved. There are known interventions that can reduce the burden of nosocomial infections in ICUs even in the resource-limited setting [15–19]. There needs to be improved recording and ongoing surveillance in order to monitor the burden of infections and evaluate interventions that can prevent nosocomial infections and reduce the risk of outbreaks.

## Figures and Tables

**Box 1:**
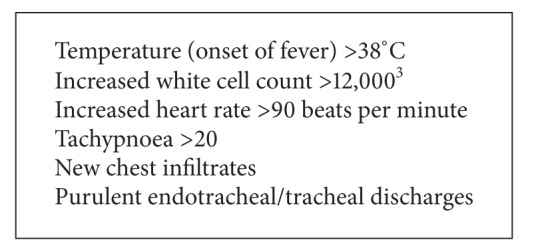
Indicators of a systemic inflammatory response.

**Table 1:** Characteristics of bacteriologically-confirmed nosocomial infections from an adult intensive care unit 2011-2012.

	2011 *N* = 58	2012 *N* = 56	Total *N* = 114
Age			
15–30 yrs	15 (26)	14 (25)	29
31–60 yrs	30 (52)	29 (52)	59
61–100 yrs	12 (21)	12 (21)	24
Unknown	—	2 (4)	2
Sex			
Male	38 (66)	34 (60)	72
Female	19 (33)	21 (38)	40
Unknown		2 (4)	2
Admission status			
Direct admission	15 (26)	19 (34)	34
Transferred	43 (74)	36 (64)	79
Unknown		1 (2)	1
Length of stay			
1–7 days	18 (31)	8 (14)	26
8–14 days	20 (34)	22 (39)	42
15–21 days	10 (17)	7 (13)	17
22–31 days	7 (12)	11 (20)	18
31 days and more	2 (3)	7 (13)	9
Unknown	1 (2)	1 (2)	2
Mechanical Ventilation			
Ventilated	57 (98)	54 (96)	111
Not ventilated	0	1 (2)	1
Unknown	1 (2)	1 (2)	2
Patient outcomes			
Transferred from ICU	35 (60)	23 (41)	58
Deceased	16 (28)	22 (39)	38
Unknown	7 (12)	11 (20)	18

**Table 2:** Distribution of bacterial pathogens associated with nosocomial infection in relation to site of specimen.

Pathogen	Site of pathogen				Total *N* = 437
	Respiratory tract *N* = 125	Blood *N* = 122	Surgical site *N* = 102	Urinary tract *N* = 88	
*Klebsiella pneumoniae* (ESBL producing^#^)	34 (27%)	21 (17%)	18 (18%)	21 (24%)	94 (22%)
*Acinetobacter* species	33 (26%)	21 (17%)	18 (18%)	20 (23%)	92 (21%)
*Pseudomonas aeruginosa *	24 (19%)	11 (9%)	25 (25%)	13 (15%)	73 (17%)
*Enterobacter *species	8 (6.4%)	9 (7%)	11 (11%)	5 (6%)	33 (8%)
Coagulase-negative *Staphylococci *	1 (0.8%)	20 (16%)	1 (0.9%)	8 (9%)	30 (7%)
*Escherichia coli *	2 (1.6%)	13 (11%)	2 (2%)	5 (6%)	22 (5%)
Other *Klebsiella *	6 (4.8%)	2 (1.6%)	6 (6%)	5 (6%)	19 (4%)
Other Gram negative species	5 (4%)	6 (5%)	6 (6%)	0	17 (4%)
*Citrobacter* species	2 (1.6%)	2 (1.6%)	2 (2%)	3 (3%)	9 (2%)
MRSA^#^	1 (0.8%)	1 (0.8%)	1 (0.9%)	—	3 (0.2%)
Others	9 (7.2%)	16 (13%)	12 (12%)	8 (9%)	45 (1.1%)

^#^ESBL: extended spectrum *β*-lactamase; MRSA: methicillin resistant *Staphylococcus aureus.*
